# Ethnic differences in the prevalence of frailty in the United Kingdom assessed using the electronic Frailty Index

**DOI:** 10.1002/agm2.12083

**Published:** 2019-09-13

**Authors:** Shraddha Pradhananga, Krishna Regmi, Nasrin Razzaq, Alireza Ettefaghian, Aparajit Ballav Dey, David Hewson

**Affiliations:** ^1^ Institute for Health Research University of Bedfordshire Luton UK; ^2^ Business Intelligence Department AT Medics Ltd London UK; ^3^ Department of Geriatric Medicine All India Institute of Medical Sciences New Delhi India

**Keywords:** aged, ethnic groups, frailty

## Abstract

**Objective:**

There have been few studies in which the prevalence of frailty of different ethnic groups has been assessed in multiethnic countries. The aim of this study was to evaluate the prevalence of frailty in different ethnic groups in the United Kingdom.

**Methods:**

Anonymized electronic health records (EHR) of 13 510 people aged 65 years and over were extracted from the database of a network of general practitioners, covering 16 clinical commissioning groups in London. Frailty was determined using the electronic Frailty Index (eFI), which was automatically calculated using EHR data. The eFI was used as a categorical variable with fit and mild frailty grouped together, and moderate and severe frailty grouped as frail.

**Results:**

The overall prevalence of frailty was 18.1% (95% confidence interval [CI], 17.4%‐18.9%). The prevalence of frailty increased with age (odds ratio [OR], 1.11; 95% CI, 1.10‐1.12) and body mass index (BMI; OR, 1.05; 95% CI, 1.04‐1.06). The highest prevalence of frailty was observed for Bangladeshis, with 32.9% classified as frail (95% CI, 29.2‐36.7); and the lowest prevalence of 14.0% (95% CI, 12.6‐15.5) was observed for the Black ethnic group. Stepwise logistic regression retained ethnicity, age, and BMI as predictors of frailty.

**Conclusion:**

This pilot study identified differences in the prevalence of frailty between ethnic groups in a sample of older people living in London. Additional studies are warranted to determine the causes of such differences, including migration and socioeconomic status. It would be worthwhile carrying out a validation study of the eFI in different ethnic populations.

## 1 INTRODUCTION

Frailty is a geriatric condition that is characterized by loss of reserves of energy, physical ability, cognition, and health due to a progressive age‐related decline in multiple physiologic systems.^1^ The consequence of frailty is a decreased capacity to respond to additional stressors, which leads to increased rates of falls, disability, hospitalization or institutionalization, or even death.^2^ Frailty is a dynamic process with transitions in both reversibility and disability even within short time periods and early detection is essential to plan the care needed to maintain health or slow down the negative effects of frailty.^3^ The most common approaches used to identify frailty are: (i) the Fried Frailty Phenotype (FFP) model^4^; and (ii) the Frailty Index (FI), otherwise known as the accumulation of deficits model.^5^ The FFP focuses on physical characteristics, with five criteria—shrinking, weakness, poor endurance, slowness, and slow activity—and classifies people according to the number of indicators into robust, pre‐frail, and frail. In contrast, the accumulation of deficit model focuses on multiple factors named “health deficits,” which are signs and symptoms of disease, laboratory measures, or disability.^6^

The prevalence of frailty varies widely, depending on the assessment methods and the population studied. The prevalence of frailty reported in the English Longitudinal Study of Ageing was 14%, with prevalence rising to 65% in those aged over 90 years.^7^ In a recent multinational study using the FI, the lowest rate of frailty was reported in China (13%), while the highest rate was observed in India (55%).^8^ The prevalence of frailty in this study was influenced by sex and socioeconomic status, with people with higher levels of education and wealth and males less likely to be frail. The prevalence of frailty is highly dependent on a complex interplay of factors, such as age, sex, lifestyle, comorbidities, socioeconomic background, and cognitive and sensory impairment. Given the difference in life course factors among different races that could be biological, genetic, psychological, social, environmental, and the accumulation of chronic disease, the prevalence of frailty is seen to be different among different races and ethnicities.^9^ With respect to ethnicity, several studies have reported the prevalence of frailty between different ethnic groups, beginning with the original FFP study in which both Caucasians and African Americans were included in the study sample.^4^ In a follow‐up paper, it was reported that African Americans were seven times more likely to be frail than Caucasians, after adjusting for levels of obesity.^10^ The differences observed were thought to be caused by socioeconomic and sociocultural factors, with African Americans faring worse in common measures of social status and resources, which would in turn increase the risk of frailty.^11^

Differences in frailty prevalence among ethnic groups could be particularly relevant in frailty screening in multiethnic countries. For instance, the United Kingdom has a Black and Asian minority ethnic population of 11%, of which 8% are South Asians.^12^ Similar differences in socioeconomic status among different ethnic groups are also present in the United Kingdom. For instance, South Asians are more prone to adiposity due to inadequate exercise and sedentary lifestyle.^13^ Frailty is highly susceptible to excessive adiposity, which in turn reduces the ability to carry out physical activity, leading to metabolic instability.^14^ In a recent study of older South Asian women living in the UK, sociocultural factors were identified as reasons for a lower physical activity level and higher prevalence of frailty than in other populations.^15^ Ethnic differences in body mass index (BMI) thresholds for obesity for South Asians are already routinely applied,^16^ which could lead to a greater capacity to detect people at risk of type 2 diabetes.^17^

To this point, there has been no study of the prevalence of frailty in the UK in which ethnicity has been taken into account. The recent adoption by the National Health Service, England, of the electronic version of the FI (eFI)^18^ could make such a study straightforward. The eFI uses data from electronic health record (EHR) systems that have records of multiple patient characteristics that are used to calculate the eFI using 36 deficits, with the ratio of deficits used to identify and grade severity of frailty.^18^ It is estimated that the implementation of the eFI by general practitioners in the UK using the EHR systems EMISweb and SystmOne would cover 90% of the total population of older English people.^19^

Accordingly, the aim of this study was to determine the prevalence of frailty in different ethnic groups in the UK in order to determine whether differences exist, in which case a more in‐depth study would be warranted to determine the reasons for any differences in frailty prevalence.

## 2 METHODS

### 2.1 Research design

This was a cross‐sectional study in partnership with AT Medics, which is the largest provider of primary care in London, UK. The AT Medics database contains data from a network of 16 clinical commissioning groups, covering a total of over 250 000 patients. The AT Medics EHR uses SNOMED clinical health‐care terminology (International Health Terminology Standards Development Organisation, London, UK), which is the internationally recognized standard. This database contains over 340 000 fields that can be used to enter medical data, all of which can be extracted for analysis (see http://snomed.org/eg for the web browsable version). Ethical approval for this secondary research study was obtained from the Institute for Health Research Ethics Committee (IHREC) at the University of Bedfordshire (IHREC907).

### 2.2 Participants

The AT Medics EHR was used to produce an anonymous data sample of all people aged over 65 years. The data extraction was performed on the October 12, 2018, at which point the database contained 235 870 patient records. Only data of people aged 65 years and over were extracted, with a total of 11 789 records extracted (5.0% of the patient records). This number is sufficient to detect a correlation of 0.015 as different from zero using magnitude‐based inferences or 0.026 using statistical significance.^20^

### 2.3 Data extracted

The variables extracted from the EHR database contained demographic information (age, sex, ethnicity, height, weight) and frailty status (eFI score and/or classification). The eFI was used as a categorical variable and participants were considered to be frail if their eFI classification was moderate or severe frailty. A proxy for socioeconomic status was used based on the geographical location of each participant. Postcodes were used to determine the Index of Multiple Deprivation (IMD), which is a weighted indicator based on seven indices, including health and disability, education, and employment, for small geographical areas in England and has been used as a proxy of socioeconomic status in health research.^21^, ^22^ In addition to the IMD, the Income Deprivation Affecting Older People Index (IDAOPI), which is a subset of the income component of the IMD for people aged over 60 years, was also used as a covariate. For both the IMD and the IDAOPI, deciles were used rather than the individual ranks of each geographical area. All data used were de‐identified at the point of extraction from the EHR to ensure participant anonymity, including hashed postcodes that were replaced by Lower Super Output Areas, from which IMD and IADOPI were obtained, and patient identification numbers.

The ethnicity data provided in the database included over 100 different ethnic classifications. These classifications were then grouped into five broad categories based on those recommended by the Office for National Statistics (ONS)^23^ of South Asian, Black, Mixed, White, and Other. The ONS categorization uses South Asian to refer to the people from India, Pakistan, and Bangladesh.^12^ Any Asian ethnicities that were not South Asian (eg, Chinese) were classified as Other. Only the results for South Asian, Black, and White participants are reported in the evaluation due to low participant numbers in other groups. An additional evaluation of the South Asian ethnic group compared Bangladeshi, Indian, and Pakistani ethnic groups, which are the three largest Asian population groups in the UK.^12^

### 2.4 Data analysis

The rates of frailty by ethnic group, age group, and sex were expressed as proportion ratios (PRs) for the appropriate population, as shown below:

(1) $$\text{PR}\;=\;\frac{x_{1}/n_{1}}{x_{2}/n_{2}},$$

where *x*_1_ and *x*_2_ are the number of frail people in the two populations (1 and 2) being compared, and n_1_ and n_2_ are the total number of people in each population.

Differences in proportions between groups were expressed as ratios, with 95% confidence intervals (CIs) for these ratios reported.^24^ CIs were calculated for all proportions by calculating the standard error of the natural logarithm of PR, which approximates a normal distribution^25^:

(2) $$\text{SE}\;\ln\;\left(\text{PR}\right)=\sqrt{\frac{1}{x_{1}}}-\frac{1}{n_{1}}+\frac{1}{x_{2}}-\frac{1}{n_{2}},$$

where *x*_1_ and *x*_2_ are the number of frail people in the two populations (1 and 2) being compared and n_1_ and n_2_ are the total number of people in each population.

The 95% CIs for ln (PR) can be expressed as:

(3) $$\text{PR}\;\pm \;e^{1.96\;\mathrm{SE}\;\ln\;(\mathrm{PR})},$$

where 1.96 corresponds to the *Z*‐score for a 95% CI.

The Kolmogorov–Smirnov test was used to test data for normality. Both the IMD and the IDAOPI deciles were not normally distributed, therefore bias‐corrected and accelerated bootstrapping was used for these variables, with data reported as means and 95% confidence limits.^26^ Results of chi‐square tests were reported to confirm statistical significance, with *P*‐values adjusted using the Bonferroni method for all post hoc tests. Stepwise logistic regression was used to provide adjusted estimates of the odds ratios (ORs) for patients being frail, with ethnic group, age, sex, BMI, IMD, and IDAOPI entered in the model. All statistical analyses were performed using IBM SPSS Statistics (Version 25).

## 3 RESULTS

### 3.1 Participants

Complete data were available for sex and age; however, all other variables had some missing data. Some postcodes were erroneous, with no match in the IMD database for 47 participants (0.4%), who were not included in the analysis. Ethnicity was not specified for 861 participants (7.3%), with these participants removed from all subsequent analyses, except for a confirmation of differences in frailty percentage with the overall dataset.

Of the remaining participants, 5640 were White (47.8%), 2239 were South Asian (19.0%), 2216 were Black (18.8%), 534 were from other ethnic groups (4.5%), with 299 people of mixed ethnicity (2.5%). The characteristics of the participants by ethnicity and sex are shown in Table 1.

**Table 1:** Characteristics of the participants

Ethnic group	Sex	Age (years)	Height (m)	Weight (kg)	BMI (kg/m^2^)
Black	Female	74.3 ± 6.7	1.59 ± 0.07	77.3 ± 16.6	30.4 ± 6.3
	Male	74.1 ± 7.2	1.70 ± 0.07	80.3 ± 14.7	27.5 ± 4.6
Other	Female	73.0 ± 7.5	1.55 ± 0.07	65.3 ± 15.8	27.2 ± 6.2
	Male	72.6 ± 6.4	1.67 ± 0.07	75.8 ± 15.4	27.2 ± 5.0
South Asian	Female	73.4 ± 7.1	1.51 ± 0.06	63.2 ± 13.9	27.4 ± 5.7
	Male	73.3 ± 6.7	1.66 ± 0.07	71.8 ± 12.8	25.9 ± 4.2
White	Female	75.2 ± 7.7	1.59 ± 0.07	70.8 ± 16.9	28.2 ± 6.4
	Male	73.6 ± 6.9	1.72 ± 0.07	83.4 ± 17.3	28.0 ± 5.5

Note

Data are means ± SDs.

The participants in the study were towards the lower end of the scale for both IMD and IDAOPI, which indicates participants were from deprived geographical areas. The bootstrapped mean for the IMD decile was 3.40 (95% CI, 3.37‐3.43), while the IDAOPI decile was 2.54 (95% CI, 2.50‐2.58). Means and 95% CIs for IMD and IDAOPI for all ethnic groups are shown in Table 2. There were significant differences among ethnic groups for both IMD and IDAOPI. With respect to IMD, lower values were identified for Bangladeshis, while the highest values were observed for Indians. When IDAOPI is considered, the lowest values were again for Bangladeshis, with the highest values for White participants.

**Table 2:** IMD and IDAOPI by ethnicity

Ethnicity	n	IMD	IDAOPI
Black	2209	2.80 (2.76‐2.86)*	1.85 (1.80‐1.91)*
Bangladeshi	607	2.35 (2.25‐2.44)*	1.29 (1.23‐1.35)*
Indian	853	4.37 (4.26‐4.49)*	2.75 (2.65‐2.86)
Pakistani	315	3.60 (3.45‐3.76)	2.23 (2.09‐2.39)*
White	5620	3.56 (3.50‐3.61)	2.94 (2.87‐3.00)

Note

Data are bootstrapped means and 95% confidence intervals.

Abbreviations: IDAOPI, Income Deprivation Affecting Older People Index; IMD, Index of Multiple Deprivation.

*Significant difference from White ethnicity (*P* < .05).

**Figure 1:**
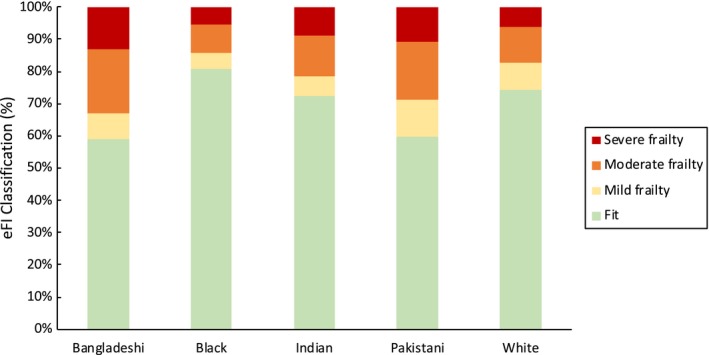
Prevalence of frailty by ethnicity. Data are percentages of people in each frailty category, as classified by the electronic Frailty Index (eFI)

The classification of participants into the four categories of frailty (fit, mild, moderate, severe) for all ethnic groups is shown in Figure 1. When moderate and severe frailty categories were combined, the overall prevalence of frailty in the population sampled was 18.1% (95% CI, 17.4‐18.9).

The prevalence of frailty for each ethnic group is shown in Table 3. The greatest prevalence of frailty was in South Asians, with Bangladeshis having the highest prevalence of frailty, followed by Pakistanis and Indians. Stepwise logistic regression retained age, BMI, and ethnicity in the model. With respect to age, there was an increased likelihood of being frail for older people (OR, 1.11; 95% CI, 1.10‐1.12; χ^2^ = 715.86, *df* = 1, *P* < .001), while those with higher BMI also had an increased likelihood of being frail (OR, 1.05; 95% CI, 1.04‐1.06; χ^2^ = 84.03, *df* = 1, *P* < .001). The ORs from the logistic regression for ethnicity are shown in Table 3. When the White ethnic group was taken as the reference, there was an increased likelihood of being frail for Bangladeshis, Pakistanis, and Indians. In contrast, there was a decrease in the likelihood of being frail for those of Black ethnicity.

**Table 3:** Prevalence of frailty by ethnicity

Ethnicity	n	Frailty prevalence	Logistic regression
Black	2209	14.0 (12.6‐15.5)	OR, 0.78 (0.63‐0.92), χ^2^ = 11.87, *df* = 1, *P* = .001
Bangladeshi	607	32.9 (29.2‐36.7)	OR, 3.01 (2.81‐3.20), χ^2^ = 120.56, *df* = 1, *P* < .001
Indian	853	21.6 (18.8‐24.3)	OR, 1.66 (1.47‐1.85), χ^2^ = 27.08, *df* = 1, *P* < .001
Pakistani	315	28.6 (23.6‐33.6)	OR, 2.44 (2.17‐2.71), χ^2^ = 41.89, *df* = 1, *P* < .001
White	5620	17.2 (16.2‐18.2)	—

Note

Frailty prevalence values are bootstrapped means and 95% confidence intervals; OR values in parentheses are 95% confidence intervals; ORs are calculated compared to White ethnicity.

Abbreviation: OR, odds ratio.

## 4 DISCUSSION

The present study is the first reported attempt at analyzing the prevalence of frailty among different ethnic groups in England. In a sample of older people living in London, South Asians were more likely to be frail than all other ethnic groups, with people of Black ethnicity least likely to be frail. The difference in frailty prevalence for South Asians when compared to Whites equates to a small effect.^27^ When differences were examined within the South Asian group, older Bangladeshis were more likely to be frail than older Indians and Pakistanis. Other studies have also reported differences in frailty prevalence among ethnic groups, with Fried et al^4^ reporting that African Americans were twice as likely to be frail as Caucasians. These findings were expanded upon in a later article in which adjusted ORs for frailty were 4.4 for nonobese men (95% CI, 2.4‐8.1) and 4.4 for nonobese women (95% CI, 2.5‐7.8) when African Americans were compared to Caucasian Americans.^10^ Similar differences have also been reported between Americans of Mexican ethnicity and European ethnicity, with older people of Mexican ethnicity 50% more likely to be frail than those of European ethnicity.^28^

In the present study, frailty prevalence was influenced by age and BMI, with older people more likely to be frail and people with greater BMI more likely to be frail. When the effects of age, sex, BMI, IMD, and IDAOPI were included as covariates, differences in frailty prevalence between ethnic groups persisted, with South Asians more likely to be frail, although the only covariates retained in the model were age and BMI. The differences in the risk of frailty for older people have been well documented in previous studies, with older people more likely to be frail.^29^

The sample in the present study came from a single network of general practices in London, England. As such, this study is not purported to be representative of the population in England but does represent an initial attempt to assess the link between frailty and ethnicity in the UK. It should be acknowledged that the population is different in London to the rest of England, being substantially younger and of greater ethnic diversity that in other regions of the country. The estimated total population of London as of July 2017 was 8 825 000, of which those aged over 65 years comprised only 11.8%, compared to the national average of 18.2%.^30^ Furthermore, the population sample for the present study contained only 5.0% of older people. Although this means that the data in the present study are not representative of the entire country, this study does provide an initial evaluation of the prevalence of frailty in different ethnic groups in the UK. In a recent report of the prevalence of frailty in the UK using the eFI, 12.9% of people were classified as moderately or severely frail.^31^ This disparity between frailty prevalence in the two populations means it would be worthwhile to extend the study to include a more representative sample of older people from different parts of the country.

In the present study, geographical area was used to provide an estimate of socioeconomic status. Although differences were identified in both IMD and IDAOPI between ethnic groups, when all variables were entered into a logistic regression, socioeconomic status was not retained in the model. This could be explained by differences between frailty prevalence and IMD, with Black ethnicity having the lowest frailty prevalence despite having a low IMD. Likewise, the Indian participants had higher IMD than the White participants, but also had a higher prevalence of frailty.

If a more detailed study was to be undertaken, it would be imperative to include additional confounding factors, such as more accurate indicators of socioeconomic status, physical activity levels, and diet. Many of these factors would be likely to vary substantially among different ethnic groups. For instance, previous studies have reported the highly sedentary behavior of South Asian and other older migrant women, leading to high prevalence of frailty.^15^

The effect of migration might also be worth investigating. In a previous study, Brothers et al^32^ identified differences in frailty prevalence in Europe, with migrants from low‐ and middle‐income countries more likely to be frail than migrants from high‐income countries and Europeans. The dataset used in the present study did not contain birthplace information, meaning that participants could have been first‐generation migrants or second‐generation migrants born in the UK. Future work should address this issue, as migration, ethnicity, and health are important issues that need to be addressed.^33^ Indeed, a universal strategy to effect health disparities caused by migration is a key priority due to the major effects caused by migration on both environment and lifestyle.^34^

The major limitations of this study were that it was a nonrepresentative cross‐sectional study from one area of London. Furthermore, the study was based on electronic health records, most of which did not contain sufficient information on potential confounding variables. However, despite these limitations, the present study presents an important first step in identifying potential differences in the prevalence of frailty in different ethnic groups in the UK. Additional work is needed to identify the links between other risk factors for frailty and ethnicity, ideally including a more representative sample of the UK population.

## CONFLICTS OF INTEREST

The authors declare no conflicts of interest.

## References

[1] Rockwood K , Song XW , MacKnight C , et al. A global clinical measure of fitness and frailty in elderly people. Can Med Assoc J. 2005;173(5):489‐495. 16129869 10.1503/cmaj.050051 PMC1188185

[2] Xue QL . The frailty syndrome: definition and natural history. Clin Geriatr Med. 2011;27(1):1‐15. 21093718 10.1016/j.cger.2010.08.009 PMC3028599

[3] Bentur N , Sternberg SA , Shuldiner J . Frailty transitions in community dwelling older people. Isr Med Assoc J. 2016;18(8):449‐453. 28471574

[4] Fried LP , Tangen CM , Walston J , et al. Frailty in older adults: evidence for a phenotype. J Gerontol A Biol Sci Med Sci. 2001;56(3):M146‐M157. 11253156 10.1093/gerona/56.3.m146

[5] Mitnitski AB , Mogilner AJ , Rockwood K . Accumulation of deficits as a proxy measure of aging. ScientificWorldJournal. 2001;1:323‐336. 12806071 10.1100/tsw.2001.58 PMC6084020

[6] Rockwood K , Hogan DB , MacKnight C . Conceptualisation and measurement of frailty in elderly people. Drugs Aging. 2000;17(4):295‐302. 11087007 10.2165/00002512-200017040-00005

[7] Gale CR , Cooper C , Sayer AA . Prevalence of frailty and disability: findings from the English Longitudinal Study of Ageing. Age Ageing. 2015;44(1):162‐165. 25313241 10.1093/ageing/afu148 PMC4311180

[8] Biritwum RB , Minicuci N , Yawson AE , et al. Prevalence of and factors associated with frailty and disability in older adults from China, Ghana, India, Mexico. Russia and South Africa. Maturitas. 2016;91:8‐18. 27451316 10.1016/j.maturitas.2016.05.012

[9] Rodriguez‐Mañas L , Fried LP . Frailty in the clinical scenario. Lancet. 2015;385(9968):e7‐e9. 25468154 10.1016/S0140-6736(14)61595-6

[10] Hirsch C , Anderson ML , Newman A , et al. The association of race with frailty: the Cardiovascular Health Study. Ann Epidemiol. 2006;16(7):545‐553. 16388967 10.1016/j.annepidem.2005.10.003

[11] Kaufman JS , Cooper RS . RE: Hirsch C, Anderson ML, Newman A, Kop W, Jackson S, Gottdiener J, et al., for the Cardiovascular Health Study Research Group. The association of race with frailty: the Cardiovascular Health Study. Ann Epidemiol. 2006;16:545‐553. Ann Epidemiol. 2007;17(2):157‐158. 17174569 10.1016/j.annepidem.2006.08.003

[12] Office for National Statistics: Ethnicity and National Identity in England and Wales 2011. http://www.ons.gov.uk/peoplepopulationandcommunity/culturalidentity/ethnicity/articles/ethnicityandnationalidentityinenglandandwales/2012-12-11. Published December 11, 2012. Accessed September 9, 2018.

[13] Fernando E , Razak F , Lear SA , Anand SS . Cardiovascular disease in South Asian migrants. Can J Cardiol. 2015;31(9):1139‐1150. 26321436 10.1016/j.cjca.2015.06.008

[14] Starr KNP , McDonald SR , Bales CW . Obesity and physical frailty in older adults: a scoping review of lifestyle intervention trials. J Am Med Dir Assoc. 2014;15(4):240‐250. 24445063 10.1016/j.jamda.2013.11.008 PMC4023554

[15] Castaneda‐Gameros D , Redwood S , Thompson JL . Physical activity, sedentary time, and frailty in older migrant women from ethnically diverse backgrounds: a mixed‐methods study. J Aging Phys Act. 2018;26(2):194‐203. 28605284 10.1123/japa.2016-0287

[16] Vasudevan D , Stotts AL , Mandayam S , Omegie LA . Comparison of BMI and anthropometric measures among South Asian Indians using standard and modified criteria. Public Health Nutr. 2011;14(5):809‐816. 21247513 10.1017/S1368980010003307

[17] Hudda MT , Nightingale CM , Donin AS , et al. Patterns of childhood body mass index (BMI), overweight and obesity in South Asian and black participants in the English National Child Measurement Programme: effect of applying BMI adjustments standardising for ethnic differences in BMI‐body fatness associations. Int J Obes. 2018;42(4):662‐670. 10.1038/ijo.2017.272 PMC5815501 29093538

[18] Clegg A , Bates C , Young J , et al. Development and validation of an electronic frailty index using routine primary care electronic health record data. Age Ageing. 2016;45(3):353‐360. 26944937 10.1093/ageing/afw039 PMC4846793

[19] Clegg A , Bates C , Young J , Teale E , Parry J . Development and validation of an electronic frailty index using existing primary care health record data. Age Ageing. 2014;43(suppl 2):ii19. 10.1093/ageing/afx001 PMC6016616 28100452

[20] Hopkins WG . Estimating sample sizes for magnitude‐based inferences. Sportscience. 2006;10:63‐70.

[21] Noble M , Wright G , Smith G , Dibben C . Measuring multiple deprivation at the small‐area level. Environ Plan A. 2006;38(1):169‐185.

[22] Auguet OT , Betley JR , Stabler RA , et al. Evidence for community transmission of community‐associated but not health‐care‐associated methicillin‐resistant *Staphylococcus aureus* strains linked to social and material deprivation: spatial analysis of cross‐sectional data. PLoS Med. 2016;13(1):24. 10.1371/journal.pmed.1001944 PMC4727805 26812054

[23] Office for National Statistics. Ethnic group, national identity and religion: a guide for the collection and classification of ethnic group, national identity and religion data in the UK. http://www.ons.gov.uk/methodology/classificationsandstandards/measuringequality/ethnicgroupnationalidentityandreligion. Published 2011. Accessed September 10, 2018.

[24] Gardner MJ , Altman DG . Confidence‐intervals rather than P values: estimation rather than hypothesis testing. Br Med J. 1986;292(6522):746‐750. 3082422 10.1136/bmj.292.6522.746 PMC1339793

[25] Rothman KJ . Epidemiology: An Introduction, 2nd edn New York, NY: Oxford University Press; 2012.

[26] Kelley K . The effects of nonnormal distributions on confidence intervals around the standardized mean difference: bootstrap and parametric confidence intervals. Educ Psychol Meas. 2005;65(1):51‐69.

[27] Hopkins WG , Marshall SW , Batterham AM , Hanin J . Progressive statistics for studies in sports medicine and exercise science. Med Sci Sports Exerc. 2009;41(1):3‐12. 19092709 10.1249/MSS.0b013e31818cb278

[28] Espinoza SE , Hazuda HP . Frailty in older Mexican‐American and European‐American adults: is there an ethnic disparity? J Am Geriatr Soc. 2008;56(9):1744‐1749. 18662198 10.1111/j.1532-5415.2008.01845.x

[29] Syddall H , Roberts HC , Evandrou M , Cooper C , Bergman H , Sayer AA . Prevalence and correlates of frailty among community‐dwelling older men and women: findings from the Hertfordshire Cohort Study. Age Ageing. 2010;39(2):197‐203. 20007127 10.1093/ageing/afp204 PMC3546311

[30] Office for National Statistics. Population estimates for the UK, England and Wales, Scotland and Northern Ireland: mid-2017. http://www.ons.gov.uk/peoplepopulationandcommunity/populationandmigration/populationestimates/bulletins/annualmidyearpopulationestimates/mid2017. Published June 28th, 2018. Accessed September 10, 2018.

[31] Reeves D , Pye S , Ashcroft DM , et al. The challenge of ageing populations and patient frailty: can primary care adapt? BMJ. 2018;362:k3349. 30154082 10.1136/bmj.k3349

[32] Brothers TD , Theou O , Rockwood K . Frailty and migration in middle‐aged and older Europeans. Arch Gerontol Geriatr. 2014;58(1):63‐68. 23993266 10.1016/j.archger.2013.07.008

[33] Wickramage K , Vearey J , Zwi AB , Robinson C , Knipper M . Migration and health: a global public health research priority. BMC Public Health. 2018;18(1):987. 30089475 10.1186/s12889-018-5932-5 PMC6083569

[34] Krasnik A , Bhopal RS , Gruer L , Kumanyika SK . Advancing a unified, global effort to address health disadvantages associated with migration, ethnicity and race. Eur J Pub Health. 2018;28(suppl 1):1‐2. 10.1093/eurpub/cky046 29617992

